# Diagnostic performance of ^18^F-FDG PET/CT and whole-body diffusion-weighted imaging with background body suppression (DWIBS) in detection of lymph node and bone metastases from pediatric neuroblastoma

**DOI:** 10.1007/s12149-018-1254-z

**Published:** 2018-04-17

**Authors:** Hiroaki Ishiguchi, Shinji Ito, Katsuhiko Kato, Yusuke Sakurai, Hisashi Kawai, Naotoshi Fujita, Shinji Abe, Atsushi Narita, Nobuhiro Nishio, Hideki Muramatsu, Yoshiyuki Takahashi, Shinji Naganawa

**Affiliations:** 1 Department of Radiology, Nagoya University Graduate School of Medicine, Nagoya, Japan; 2 Department of Radiology, Toyohashi Municipal Hospital, Toyohashi, Japan; 3 Department of Radiological and Laboratory Sciences, Nagoya University Graduate School of Medicine, 1-20, Daikominami 1-chome, Higashi-ku, Nagoya, 461-8673 Japan; 4 Department of Radiological Technology, Nagoya University Hospital, Nagoya, Japan; 5 Department of Pediatrics, Nagoya University Graduate School of Medicine, Nagoya, Japan

**Keywords:** ^18^F-FDG PET/CT, Whole-body DWIBS, Neuroblastoma, Metastasis

## Abstract

**Objective:**

Recent many studies have shown that whole body “diffusion-weighted imaging with background body signal suppression” (DWIBS) seems a beneficial tool having higher tumor detection sensitivity without ionizing radiation exposure for pediatric tumors. In this study, we evaluated the diagnostic performance of whole body DWIBS and ^18^F-FDG PET/CT for detecting lymph node and bone metastases in pediatric patients with neuroblastoma.

**Methods:**

Subjects in this retrospective study comprised 13 consecutive pediatric patients with neuroblastoma (7 males, 6 females; mean age, 2.9 ± 2.0 years old) who underwent both ^18^F-FDG PET/CT and whole-body DWIBS. All patients were diagnosed as neuroblastoma on the basis of pathological findings. Eight regions of lymph nodes and 17 segments of skeletons in all patients were evaluated. The images of ^123^I-MIBG scintigraphy/SPECT-CT, bone scintigraphy/SPECT, and CT were used to confirm the presence of lymph node and bone metastases. Two radiologists trained in nuclear medicine evaluated independently the uptake of lesions in ^18^F-FDG PET/CT and the signal-intensity of lesions in whole-body DWIBS visually. Interobserver difference was overcome through discussion to reach a consensus. The sensitivities, specificities, and overall accuracies of ^18^F-FDG PET/CT and whole-body DWIBS were compared using McNemer’s test. Positive predictive values (PPVs) and negative predictive values (NPVs) of both modalities were compared using Fisher’s exact test.

**Results:**

The total numbers of lymph node regions and bone segments which were confirmed to have metastasis in the total 13 patients were 19 and 75, respectively. The sensitivity, specificity, overall accuracy, PPV, and NPV of ^18^F-FDG PET/CT for detecting lymph node metastasis from pediatric neuroblastoma were 100, 98.7, 98.9, 95.0, and 100%, respectively, and those for detecting bone metastasis were 90.7, 73.1, 80.3, 70.1, and 91.9%, respectively. In contrast, the sensitivity, specificity, overall accuracy, PPV, and NPV of whole-body DWIBS for detecting bone metastasis from pediatric neuroblastoma were 94.7, 24.0, 53.0, 46.4 and 86.7%, respectively, whereas those for detecting lymph node metastasis were 94.7, 85.3, 87.2, 62.1, and 98.5%, respectively. The low specificity, overall accuracy, and PPV of whole-body DWIBS for detecting bone metastasis were due to a high incidence of false-positive findings (82/108, 75.9%). The specificity, overall accuracy, and PPV of whole-body DWIBS for detecting lymph node metastasis were also significantly lower than those of ^18^F-FDG PET/CT for detecting lymph node metastasis, although the difference between these 2 modalities was less than that for detecting bone metastasis.

**Conclusion:**

The specificity, overall accuracy, and PPV of whole-body DWIBS are significantly lower than those of ^18^F-FDG PET/CT because of a high incidence of false-positive findings particularly for detecting bone metastasis, whereas whole-body DWIBS shows a similar level of sensitivities for detecting lymph node and bone metastases to those of ^18^F-FDG PET/CT. DWIBS should be carefully used for cancer staging in children because of its high incidence of false-positive findings in skeletons.

## Introduction

Neuroblastoma is the most common and malignant solid tumors of children [1], accounting for about 8% of all malignancy of children [2]. Metastases were detected approximately in 70% of patients at the time when they were diagnosed as neuroblastoma and metastasis was most commonly present at cortical bone and bone marrow [3]. Accurate diagnosis of tumor staging is important to determine treatment planning.

^18^F-FDG accumulates in primary and metastatic tumors of neuroblastoma, and the use of ^18^F-FDG PET or ^18^F-FDG PET/CT for the diagnosis of patients with neuroblastoma is increasing [4–8].

Whole-body “diffusion-weighted imaging with background body signal suppression” (DWIBS) which was introduced by Takahara et al. [9] in 2004 has been increasingly used, and many studies confirmed that whole-body DWIBS is a feasible clinical technique for assessment of the original and metastatic lesions of adult patients with high sensitivity and accuracy [10–14]. In the field of pediatric oncology, it has recently been demonstrated that, based on apparent diffusion coefficient (ADC) values, DWIBS can discriminate between benign and malignant abdominal mass lesions [15], between neuroblastoma and ganglioneuroma/ganglioneuroblastoma [16–18], and between neuroblastoma and Wilms tumor [19]. Change in ADC values is useful for efficiently judging response to chemotherapy in pediatric neuroblastoma [20]. There has also been a case report which proposes diagnostic value of DWIBS for detecting metastases of pediatric neuroblastoma [21]. These studies suggest that DWIBS may be a beneficial tool having high detection sensitivity without ionizing radiation exposure for pediatric tumors.

The present study was scheduled as a pilot study to assess and compare the availabilities of DWIBS and ^18^F-FDG PET/CT for detecting lymph node and bone metastases in pediatric patients with neuroblastoma. For this purpose, qualitative analysis was adopted instead of quantitative analysis so as to acquire early preliminary information for the future study.

## Materials and methods

### Subjects

This retrospective study had institutional review board approval (Approval No. 2014-0150), and the need to obtain patient informed consent for the study was waived. Subjects in this study comprised consecutive 13 patients (7 males, 6 females; mean age, 2.9 ± 2.0 years old) who were diagnosed as neuroblastoma based on pathological findings at Nagoya University Hospital between June 2012 and January 2016. All patients underwent ^18^F-FDG PET/CT, whole-body DWIBS, ^123^I-meta-iodobenzylguanidine (^123^I-MIBG) scintigraphy/SPECT-CT, bone scintigraphy/SPECT, and CT for the initial tumor staging within an interval of 20 days, 6 patients underwent them before starting the treatment, and 7 patients underwent them by at least 13 days after starting the treatment. For assessments by these imaging modalities, patients were administered with sedative orally or intravenously. For staging of patients with neuroblastoma, we used International Neuroblastoma Staging System (INSS) [22].

### ^18^F-FDG PET, CT, and PET fused with CT (^18^F-FDG PET/CT)

^18^F-FDG PET/CT examination was performed with a PET/CT scanner (Biograph 16; Siemens Healthcare, Erlangen, Germany). Patients were fasted for at least 6 h prior to intravenous administration of ^18^F-FDG at a dose of 3.7 MBq/kg body weight, and positioned head first and supine, and arms were down throughout the scanning procedure. Images were obtained from the skull to the mid-thigh 50 min after ^18^F-FDG injection. CT was performed according to a standardized protocol with the following parameters: 16-detector row, 120 kv, 100 mAs, 5 mm slice-thickness and 4 mm pitch. Patients maintained normal shallow respiration during the acquisition of CT scans. Immediately after the unenhanced CT, PET was performed in the identical transverse field of view. The acquisition time was 1.7 min per table position. The resulting PET and CT scans were coregistered automatically on the workstation.

### Magnetic resonance imaging (MRI) and whole-body “diffusion-weighted imaging with background body signal suppression” (DWIBS)

MRI was performed with 1.5-T MRI scanner (MAGNETOM Avanto; Siemens Healthcare, Erlangen, Germany or MAGNETOM Aera; Siemens Healthcare, Erlangen, Germany). Whole-body DWIBS images were obtained in the axial plane with a spin-echo single-shot echo planar imaging incorporating generalized autocalibrating partially parallel acquisitions. The parameters were as follows: TR/TE, 6600-12033/76-83 ms; matrix size, 1.66 × 1.66–2.08 × 2.08 mm^2^; slice thickness, 4.0–5.0 mm; slice gap, 4.0–6.0 mm.

STIR was used for fat suppression.

Motion probing gradient pulses were applied along three orthogonal directions with b-values of 800 s/mm^2^. Three-dimensional maximum intensity projection (3D-MIP) images were reconstructed from the axial DWIBS images.

### ^123^I-meta-iodobenzylguanidine (^123^I-MIBG) scintigraphy/SPECT-CT

^123^I-MIBG scintigraphy was performed 6 and 24 h after intravenous injection of 37–45 MBq ^123^I-MIBG (FUJI FILM RI Pharma Co., Ltd). Anterior and posterior whole-body planar images and SPECT-CT images were obtained using a dual-head gamma camera (Symbia T or T6, Siemens Healthcare, Erlangen, Germany). The low-medium energy general purpose collimator (LMEGP) was set on the SPECT equipment. Syngo MI Application 2009A was used as a reconstruction software.

### Bone scintigraphy/SPECT

^99m^Tc-bone scintigraphy was performed 3 h after intravenous injection of 200–290 MBq ^99m^Tc-HMDP (Nihon Medi-Physics Co., Ltd) or 200–290 MBq ^99m^Tc-MDP (FUJI FILM RI Pharma Co., Ltd). Anterior and posterior whole-body planar images and SPECT images were obtained using a dual-head gamma camera (Symbia T or T6, Siemens Healthcare, Erlangen, Germany). The low energy high resolution collimator (LEHR) was set on the SPECT equipment. Syngo MI Application 2009A was used as a reconstruction software.

### Image analysis

The presence of lymph node metastasis was assessed in 8 regions: right and left supraclavicular, right and left axillary, mediastinal, paraaortic, and right and left pelvic regions. The presence of bone metastasis was assessed in 17 bone segments: skull, sternum, cervical, thoracic and lumbar spines, right and left humeri, right and left scapulae, right and left clavicles, right and left ribs, right and left pelves, and right and left femurs.

For visual analysis, images were displayed on a clinical workstation that allowed interactive exploration of image data of various modalities. In whole-body DWIBS, signal intensity of skeletal muscles was used as the reference standard for the judgement of positive results. In ^18^F-FDG PET/CT, ^123^I-MIBG scintigraphy/SPECT-CT, and bone scintigraphy/SPECT, the loci where uptake was visibly higher than the activity of adjacent areas were considered uptake-positive. In CT, characteristic enlarged massive images corresponding to the sites of lymph nodes were defined as metastasis-positive, and focal or diffuse lesions of skeletons with or without deformity of cortical bones were defined as metastasis-positive. Two radiologists with 3 and 12 years of experience in nuclear medicine, respectively, independently evaluated visually the images of lesions in these modalities. Interobserver difference was overcome through discussion to reach a consensus.

### Confirmation of the presence of lymph node and bone metastases

The presence of metastasis was verified by the following definitions. In case of lymph node metastasis, both ^123^I-MIBG scintigraphy/SPECT-CT and CT must show the positive findings. In case of bone metastasis, at least 2 of the 3 modalities (^123^I-MIBG scintigraphy/SPECT-CT, bone scintigraphy/SPECT, and CT) must show the positive findings. Besides, there were fewer cases in which metastatic lesions were confirmed on the basis of pathological findings by biopsy. The absence of metastasis was verified by the fact that none of these modalities showed the positive findings. The lymph node regions and bone segments which showed the positive findings in only one modality were excluded as unsuitable to evaluate. Confirmation of the presence of lymph node and bone metastases were done by the same 2 radiologists as described above.

### Statistical analysis

McNemer’s test was used to compare the sensitivities, specificities, and overall accuracies of ^18^F-FDG PET/CT and whole-body DWIBS. Fisher’s exact test was used to compare the positive predictive values (PPVs) and negative predictive values (NPVs) of both modalities. *P* values less than 0.05 were considered statistically significant. Statistical analysis was performed using Excel 2010 (Microsoft Co., Redmond, WA, USA) with the statistical add-in software for Microsoft Excel ‘Statcel3’ (OMS Ltd, Tokyo, Japan, 2011).

## Results

### Patient profiles, the original lesions, staging (INSS), lymph node and bone metastases, treatments, and prognosis

Patient profiles, location of the original lesion, staging (INSS), and the presence of metastases in lymph nodes and bones, treatments, and prognosis are shown in Table 1. The original lesions of neuroblastoma were in adrenal glands in 10 patients, retroperitoneal regions in 2 patients, and posterior mediastinum in a patient. All the total 13 patients had stage IV (INSS). Nine of the total 13 patients had metastases in both lymph nodes and bones, 2 patients (patients 8 and 10) had metastasis in lymph nodes but no metastasis in bones, and 2 patients (patients 4 and 13) had metastasis in bones but no metastasis in lymph nodes.

**Table 1:** Patient profiles, location of the original lesion, staging (INSS), and presence of metastases in lymph nodes and bones

Patient no.	Age (years and months)	Sex	Original lesion	Stage (!NSS)	Lymph node metastasis	Bone metastasis	Treatment	Prognosis (at present)
1	2 y 9 m	F	Right adrenal gland	IV	+	+	Chemotherapy Retinoic acid	Follow-up^a^
2	1 y 11 m	M	Left adrenal gland	IV	+	+	Chemotherapy	Follow-up
3	4 y 9 m	F	Right adrenal gland	IV	+	+	Chemotherapy Retinoic acid	Follow-up
4	2 y 4 m	M	Left adrenal gland	IV	−	+	Chemotherapy Surgical excision for metastasis in skull	Follow-up
5	2 y 10 m	M	Left adrenal gland	IV	+	+	Chemotherapy	Died due to the original disease
6	1 y 7 m	F	Bilateral retroperitoneal	IV	+	+	Chemotherapy Retinoic acid	Follow-up
7	4 y 1 m	M	Right retroperitoneal	IV	+	+	Chemotherapy	Undergoing chemotherapy
8	2 y 2 m	M	Left adrenal gland	IV	+	–	Chemotherapy Retinoic acid	Follow-up
9	2 y 9 m	F	Right adrenal gland	IV	+	+	Chemotherapy	Died due to the original disease
10	2 y 8 m	M	Left adrenal gland	IV	+	–	Chemotherapy Retinoic acid	Follow-up
11	4 y 9 m	F	Left adrenal gland	IV	+	+	Chemotherapy Hematopoietic stem cell transplantation	Died due to the original disease
12	4 y 5 m	F	Right adrenal gland	IV	+	+	Chemotherapy	Undergoing chemotherapy
13	3 y 5 m	M	Right posterior mediastinum	IV	–	+	Chemotherapy Umbilical cord blood transplantation	Follow-up

*F* female, *M* male

^a^Follow-up: The original and metastatic lesions have been reduced or disappeared by the treatments and at present the patients are under observation for follow-up

Location and number of lymph node and bone metastases confirmed to be present in the total 13 patients are shown in Tables 2 and 3, respectively. The total numbers of lymph node regions and bone segments which were confirmed to have metastasis in the total 13 patients were 19 and 75, respectively. Lymph node metastases were present in paraaortic, left supraclavicular, mediastinal, and left pelvic regions (Table 2). Bone metastases were present in left and right pelves, lumbar and thoracic spines, left and right femurs, skull, left and right humeri, left and right scapulae, sternum, and right rib (Table 3).

**Table 2:** Distribution of lymph node metastases in the total 13 patients with neuroblastoma

Region	Number of metastasis		
	Left		Right
Supraclavicular	4		0
Axillary	0		0
Mediastinal		3	
Paraaortic		11	
Pelvic	1		0
Total		19	

**Table 3:** Distribution of bone metastases in the total 13 patients with neuroblastoma

Bone segment	Number of metastasis		
	Left		Right
Skull		7	
Sternum		2	
Cervical spine		0	
Thoracic spine		7	
Lumbar spine		8	
Humerus	5		4
Scapula	2		2
Clavicle	0		0
Rib	0		1
Pelvis	10		11
Femur	8		8
Total		75	

### ^18^F-FDG PET/CT and whole-body DWIBS findings of lymph nodes and bones

^18^F-FDG PET/CT and whole-body DWIBS findings of lymph nodes and bones in the total 13 patients are shown in Table 4. Ten of the total 104 lymph node regions (8 regions × 13 patients) and 38 of the total 221 bone segments (17 segments × 13 patients) were excluded as unsuitable to evaluate in the present study. Therefore, the total number of lymph node regions assessed were 94 (19 metastasis-positive; 75 metastasis-negative). And the number of bone segments assessed were 183 (75 metastasis-positive; 108 metastasis-negative). The most conspicuous feature of the results was a very high incidence of false-positive findings in whole-body DWIBS images of bone segments without metastasis (82/108, 75.9%) compared with those in ^18^F-FDG PET/CT images of them (29/108, 26.9%) (Table 4).

**Table 4:** ^18^F-FDG PET/CT and whole-body DWIBS findings of lymph nodes and bones in the total 13 patients with neuroblastoma

Modality	Findings	Number of lymph node regions		Number of bone segments	
		With metastasis 19	Without metastasis 75	With metastasis 75	Without metastasis 108
FDG-PET/CT	+ −	19 0 (false-negative)	1 (false-positive) 74	68 7 (false-negative)	29 (false-positive) 79
DWIBS	+ −	18 1 (false-negative)	11 (false-positive) 64	71 4 (false-negative)	82 (false-positive) 26

Ten of the total 124 lymph node regions (8 regions × 13 patients) and 38 of the total 221 bone segments (17 segments × 13 patients) were excluded as unsuitable to evaluate in this study

### The sensitivity, specificity, overall accuracy, PPV, and NPV of ^18^F-FDG PET/CT and whole-body DWIBS for detecting lymph node and bone metastases

The sensitivity, specificity, overall accuracy, PPV, and NPV of ^18^F-FDG PET/CT and whole-body DWIBS for detecting lymph node and bone metastases, which were calculated from the results shown in Table 4, are shown in Tables 5 and 6, respectively.

**Table 5:** Sensitivity, specificity, overall accuracy, PPV, and NPV of ^18^F-FDG PET/CT and whole-body DWIBS for detecting lymph node metastasis from pediatric neuroblastoma

Parameter	FDG-PET	DWIBS	*P* value
Sensitivity (%)	19/19 (100)	18/19 (94.7)	ns
Specificity (%)	74/75 (98.7)	64/75 (85.3)	< 0.01
Accuracy (%)	93/94 (98.9)	82/94 (87.2)	< 0.01
PPV (%)^a^	19/20 (95.0)	18/29 (62.1)	< 0.01
NPV (%)^b^	74/74 (100)	64/65 (98.4)	ns

*ns* Not significant

^a^Positive predictive value

^b^Negative predictive value

**Table 6:** Sensitivity, specificity, and overall accuracy of ^18^F-FDG PET/CT and whole-body DWIBS for detecting bone metastasis from neuroblastoma

Parameter	FDG-PET	DWIBS	*P* value
Sensitivity (%)	68/75 (90.7)	71/75 (94.7)	ns
Specificity (%)	79/108 (73.1)	26/108 (24.0)	< 0.01
Accuracy (%)	147/183 (80.3)	97/183 (53.0)	< 0.01
PPV (%)^a^	68/97 (70.1)	71/153 (46.4)	< 0.01
NPV (%)^b^	79/86 (91.9)	26/30 (86.7)	ns

*ns* not significant

^a^Positive predictive value

^b^Negative predictive value

The sensitivities of ^18^F-FDG PET/CT and whole-body DWIBS for detecting lymph node metastasis were almost equally high (100 vs. 94.7%), whereas the specificity of whole-body DWIBS for detecting lymph node metastasis was significantly lower than that of ^18^F-FDG PET/CT (85.3 vs. 98.7%). Therefore, the overall accuracy of whole-body DWIBS for detecting lymph node metastasis was significantly lower than that of ^18^F-FDG PET/CT (87.2 vs. 98.9%). PPV of whole-body DWIBS for detecting lymph node metastasis was significantly lower than that of ^18^F-FDG PET/CT (62.1 vs. 95.0%), whereas NPVs of ^18^F-FDG PET/CT and whole-body DWIBS for detecting lymph node metastasis were almost equally high (100 vs. 98.5%) (Table 5).

Similarly to the results of lymph node metastasis, the sensitivities of whole-body DWIBS and ^18^F-FDG PET/CT were almost equally high for detecting bone metastasis (94.7 vs. 90.7%). In contrast, the specificity of whole-body DWIBS for detecting bone metastasis was significantly lower than that of ^18^F-FDG PET/CT (24.0 vs. 73.1%). Therefore, the overall accuracy of whole-body DWIBS for detecting bone metastasis was significantly lower than that of ^18^F-FDG PET/CT (53.0 vs. 80.3%). PPV of whole-body DWIBS for detecting bone metastasis was significantly lower than that of ^18^F-FDG PET/CT (46.4 vs. 70.1%), whereas NPV of whole-body DWIBS for detecting bone metastasis did not differ significantly from that of ^18^F-FDG PET/CT (86.7 vs. 91.9%) (Table 6).

### The sensitivity of ^123^I-MIBG scintigraphy/SPECT-CT for detecting lymph node and bone metastases and the sensitivity of bone scintigraphy/SPECT for detecting bone metastasis

At ^123^I-MIBG scintigraphy/SPECT-CT, 19 of the total 19 lymph node regions with metastasis were uptake-positive [sensitivity: 19/19 (100%)], and 71 of the total 75 bone segments with metastasis were uptake-positive [sensitivity: 71/75 (94.7%)]. The sensitivities of ^123^I-MIBG scintigraphy/SPECT-CT for detecting lymph node and bone metastases were compatible with those of ^18^F-FDG PET/CT [19/19 (100%) and 68/75 (90.7%), respectively]. At bone scintigraphy/SPECT, 61 of the total 75 bone segments with metastasis were uptake-positive [sensitivity: 61/75 (81.3%)]. The sensitivity of bone scintigraphy/SPECT for detecting bone metastases was inferior to that of ^18^F-FDG PET/CT [68/75 (90.7%)].

### False-positive findings in lymph nodes and bones in ^18^F-FDG PET/CT and whole-body DWIBS

Location and number of false-positive findings in bone segments without metastasis in assessments of ^18^F-FDG PET/CT and whole-body DWIBS are shown in Tables 7 and 8, respectively. The number of false-positive findings in bone segments without metastasis in whole-body DWIBS was 2.86- fold larger than that in ^18^F-FDG PET/CT. There was no particular difference in the distribution of false-positive findings between ^18^F-FDG PET/CT and whole-body DWIBS (Tables 7, 8).

**Table 7:** Location and number of false-positive findings in bone segments without metastasis in ^18^F-FDG PET/CT

Bone segments	Number of false-positive findings		
	Left		Right
Skull		0	
Sternum		4	
Cervical spine		3	
Thoracic spine		2	
Lumbar spine		2	
Humerus	2		22
Scapula	3		4
Clavicle	1		1
Rib	1		1
Pelvis	1		1
Femur	1		0
Total		29	

The total number of bone segments without metastasis assessed by ^18^F-FDG PET/CT was 108

**Table 8:** Location and number of false-positive findings in bone segments without metastasis in whole-body DWIBS

Bone segments	Number of false-positive findings		
	Left		Right
Skull		1^a^	
Sternum		7	
Cervical spine		7	
Thoracic spine		5	
Lumbar spine		5	
Humerus	5		4
Scapula	7		8
Clavicle	4		5
Rib	9		8
Pelvis	2		2
Femur	2		2
Total		83	

The total number of bone segments without metastasis assessed by whole-body DWIBS was 108

^a^False-positive image was found in sphenoidal bone of skull in the patient 6 who had a metastatic lesion in left temporal bone of skull (Fig. 2)

There were more false-positive findings in lymph node regions without metastasis in the assessment of whole-body DWIBS than that in the assessment of ^18^F-FDG PET/CT (11 vs. 1). Seven false-positive findings in lymph node regions without metastasis in whole-body DWIBS were located in left axillary region and the other 4 false-positive finding were in right axillary region. One false-positive finding in lymph node regions without metastasis in ^18^F-FDG PET/CT was located in mediastinal region (data not shown).

### Example images of the original lesions, lymph node and bone metastases, and false-positive findings

**Fig. 1:**
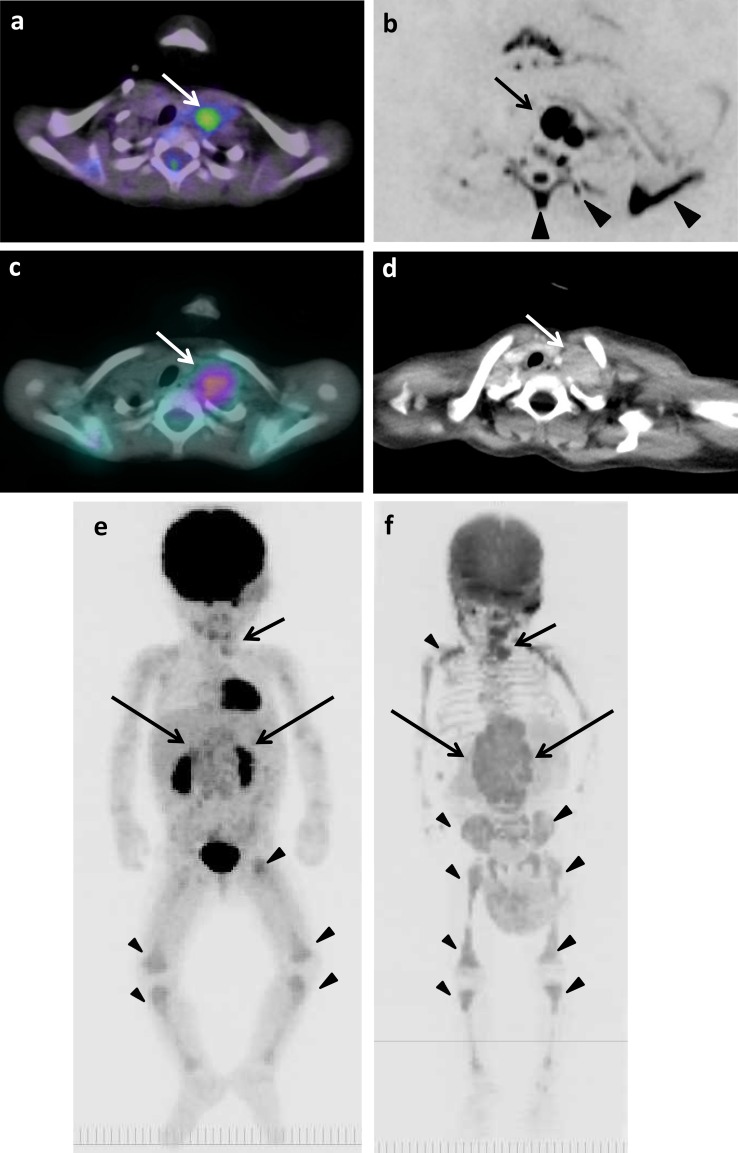
The original lesions in bilateral retroperitoneal regions, metastases in left supraclavicular lymph nodes, and false-positive images of skeletons of 1 year and 7 months old girl (patient 6). **a** ^18^F-FDG PET/CT. **b** DWIBS. **c** ^123^I-MIBG scintigraphy/SPECT-CT. **d** CT. The maximum intensity projection (MIP) of ^18^F-FDG PET (**e**) and DWIBS (**f**). Long arrows show the original lesions (**e, f**). Short arrows show metastases (**a**–**d**). Arrowheads show false-positive images in thoracic spine, left rib and scapula (**b**) and in various bone segments (**e, f**)

**Fig. 2:**
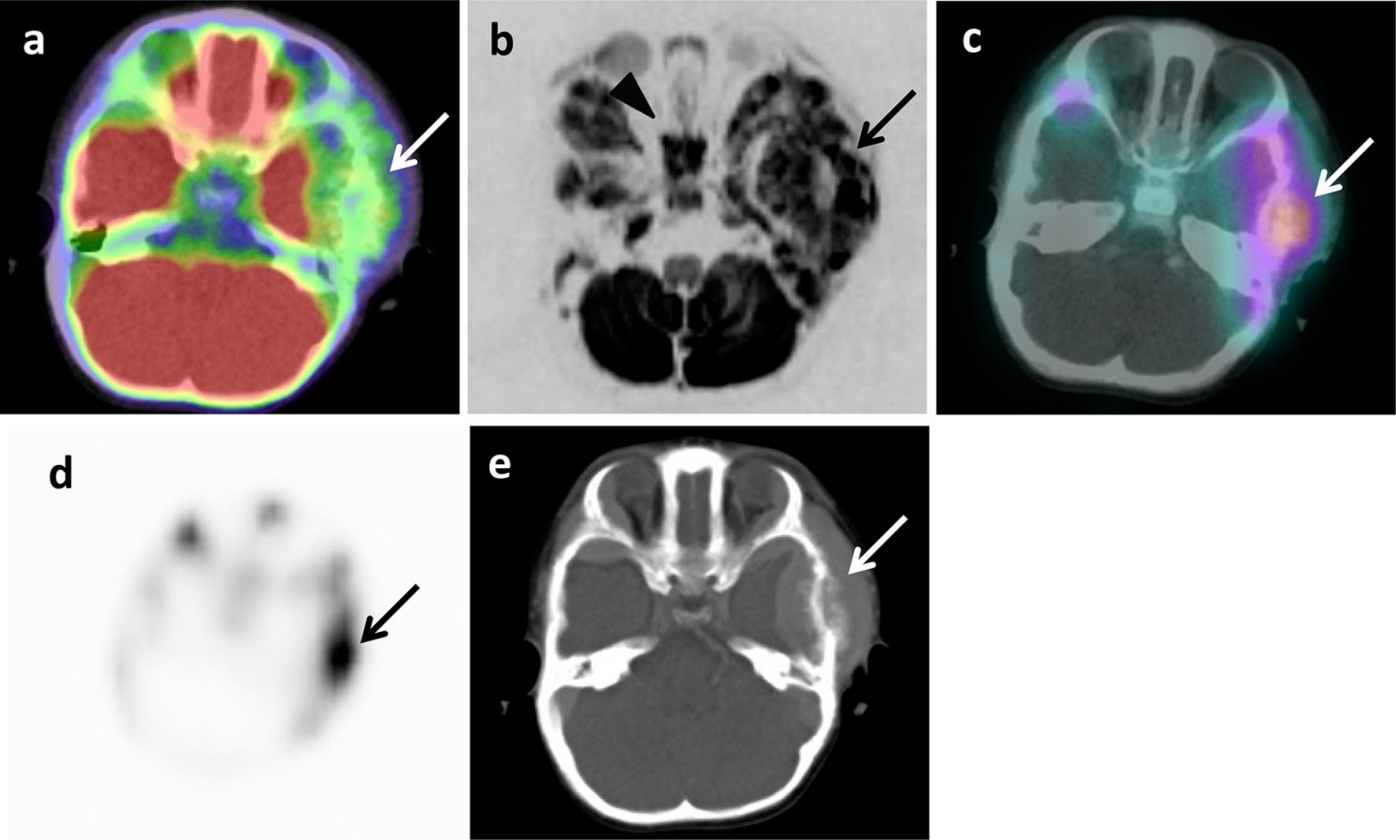
Metastasis in left temporal bone of skull of 1 year and 7 months old girl (patient 6). **a** ^18^F-FDG PET/CT. **b** DWIBS. **c** ^123^I-MIBG scintigraphy/SPECT-CT. **d** Bone scintigraphy/SPECT. **e** CT. Arrows show metastasis (**a**–**e**). Arrowhead shows false-positive images in sphenoidal bone (**b**)

**Fig. 3:**
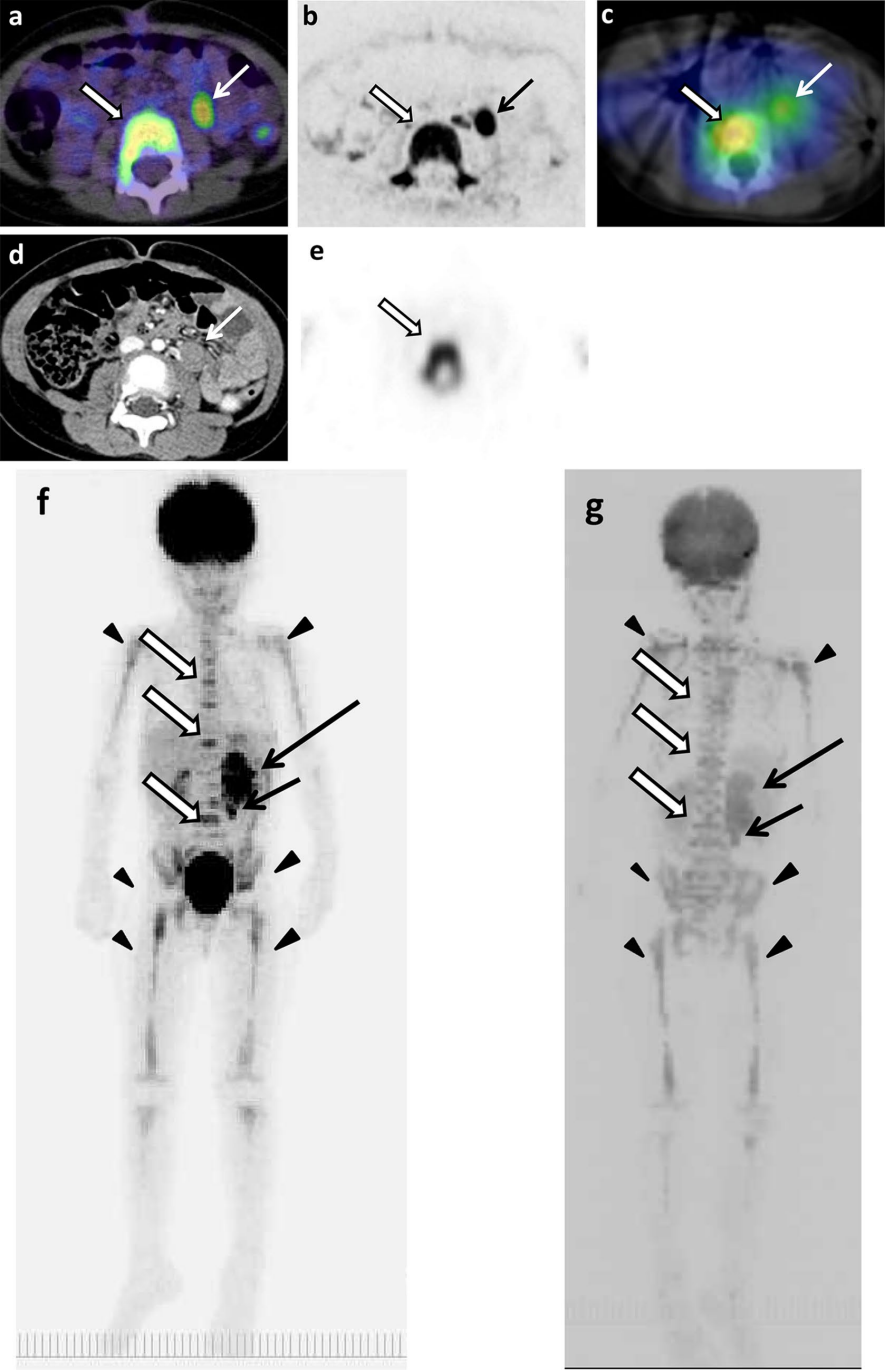
The original lesions in left adrenal gland, metastases in paraaortic lymph nodes and thoracic and lumbar spines, and false-positive images of skeletons of 4 years and 9 months old girl (patient 11). **a** ^18^F-FDG PET/CT. **b** DWIBS. **c** ^123^I-MIBG scintigraphy/SPECT-CT. **d** CT. **e** Bone scintigraphy/SPECT. The MIP of ^18^F-FDG PET (**f**) and DWIBS (**g**). Long arrows show the original lesion (**f, g**). Short arrows show lymph node metastases (**a**–**g**). Empty arrows show bone metastases (**a**–**c, e**–**g**). Arrowheads show false-positive images of various bone segments (**f, g**)

The example images of lymph node and bone metastases from neuroblastoma by ^18^F-FDG PET/CT, DWIBS, ^123^I-MIBG scintigraphy/SPECT-CT, bone scintigraphy/SPECT, and CT are shown in Figs. 1, 2 and 3. The massive metastatic lesion of temporal bone (Fig, 2a-d) accompanies deformity of bone (Fig. 2e). Diffuse intensive signal of metastasis of lumber spine (Fig. 3a–c, e) does not accompany deformity of bone (Fig. 3d). These findings suggest that bone metastasis involves both cortical bone and marrow.

**Fig. 4:**
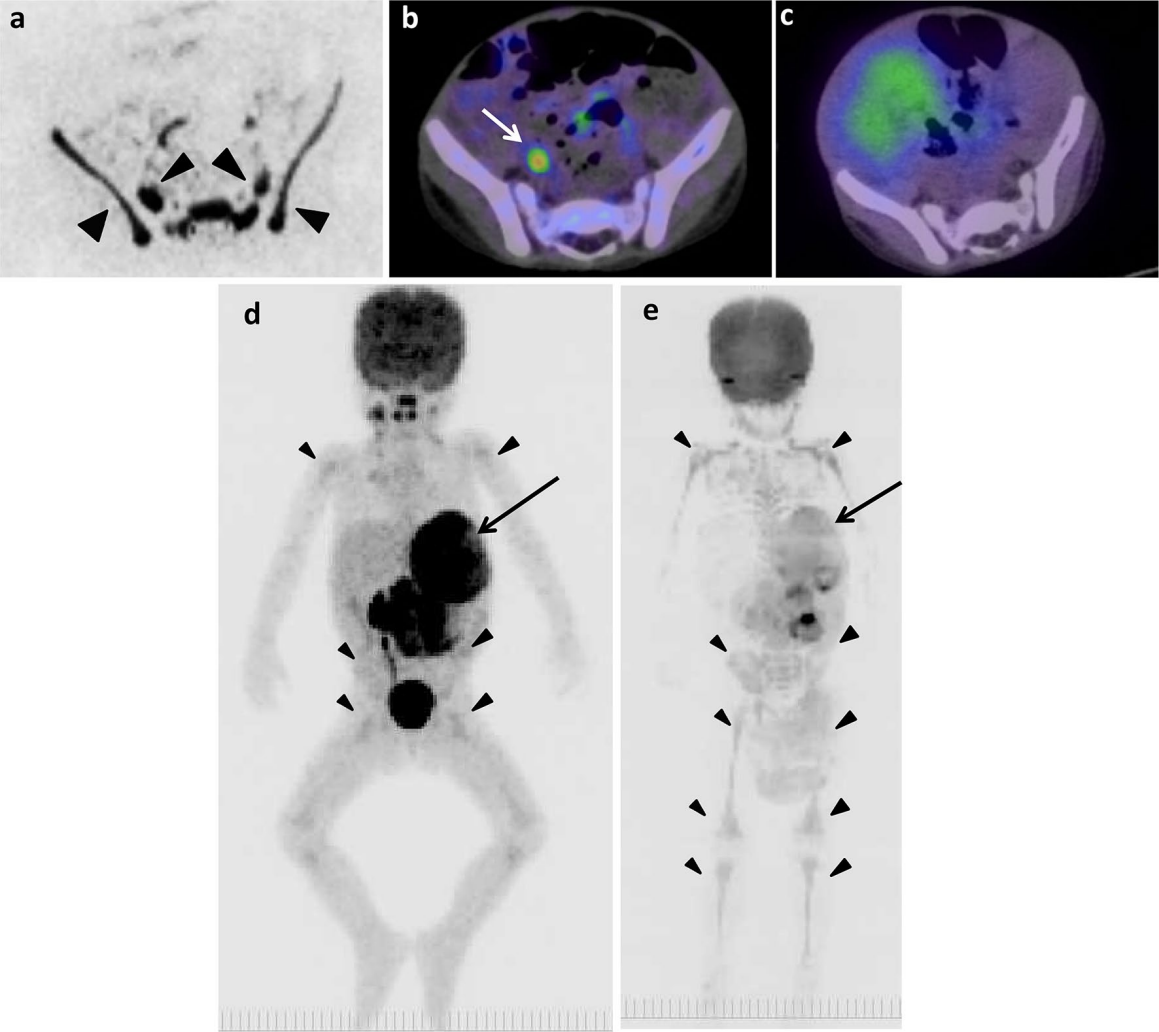
The original lesion in left adrenal gland and false-positive images of skeletons of 2 years and 2 months old boy (patient 8). **a** DWIBS. **b** ^18^F-FDG PET/CT. **c** ^123^I-MIBG scintigraphy/SPECT-CT. The MIP of ^18^F-FDG PET (**d**) and DWIBS (**e**). Long arrows show the original lesion (**d, e**). Arrowheads show false-positive images in pelvic bones (**a**) and various bone segments (**d, e**). Short arrow shows ^18^F-FDG uptake in right ureter (**b**)

**Fig. 5:**
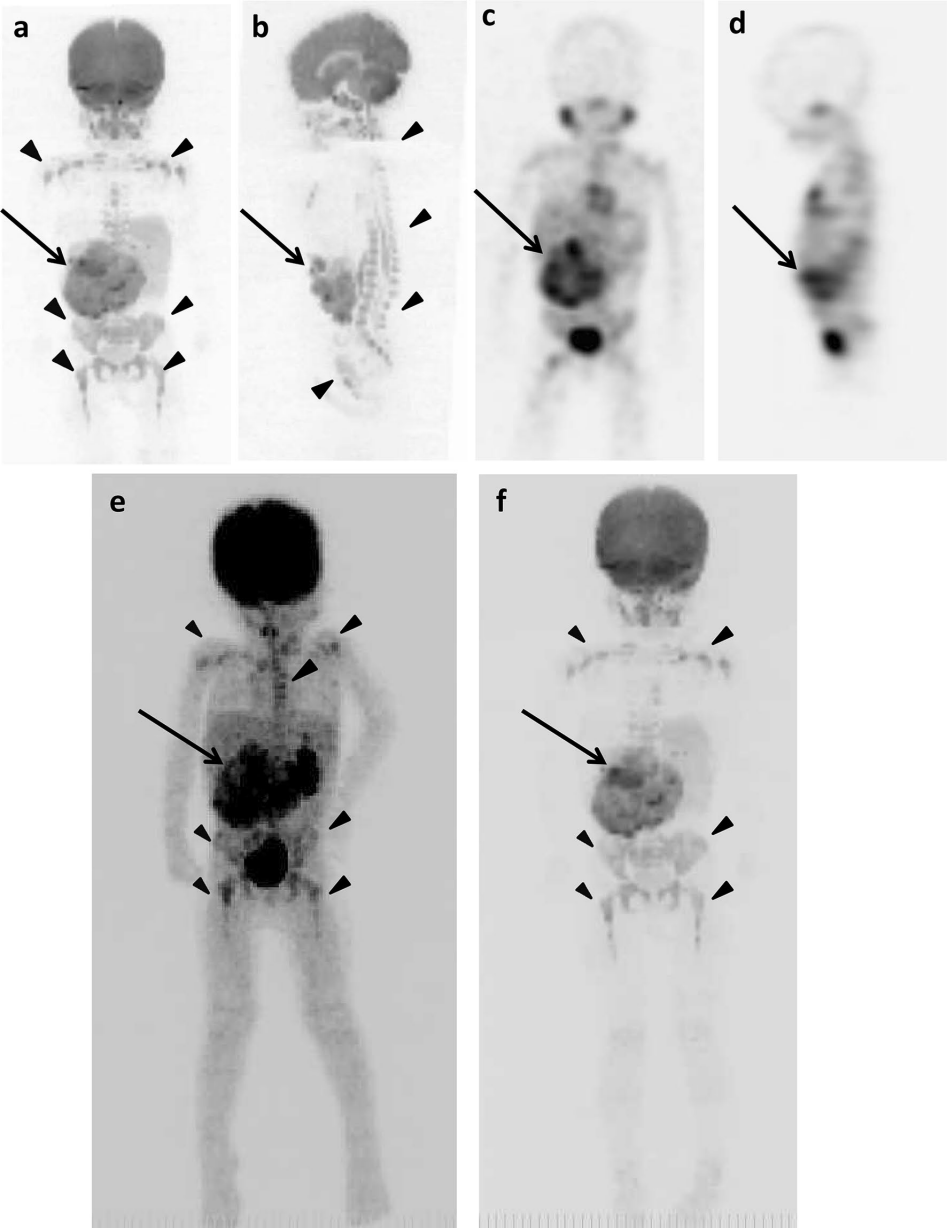
The original lesions in right adrenal gland and false-positive images in various bone segments of 2 years and 9 months old girl (patient 1). **a** DWIBS (anterior). **b** DWIBS (lateral). **c** ^123^I-MIBG scintigraphy/SPECT (anterior). **d** ^123^I-MIBG scintigraphy/SPECT (lateral). The MIP of ^18^F-FDG PET (**e**) and DWIBS (**f**). Long arrows show the original lesion (**a**–**f**). Arrowheads show false-positive images (**a, b, e, f**)

**Fig. 6:**
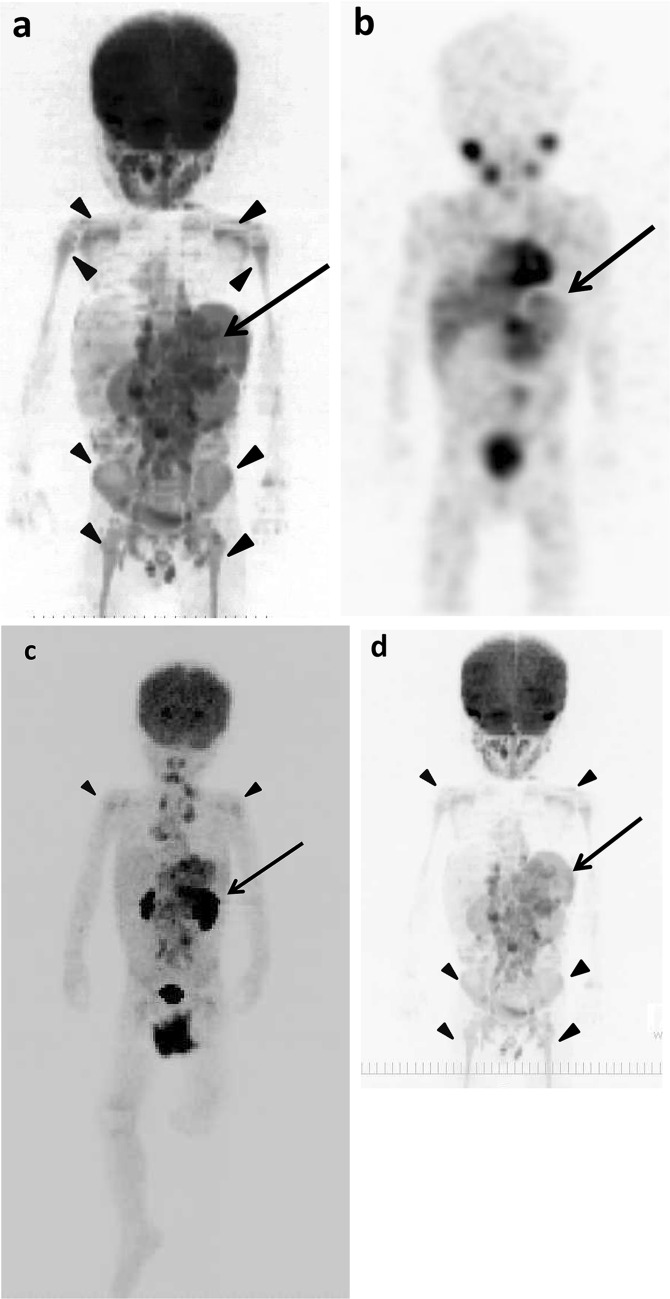
The original lesion in left adrenal gland and false-positive findings in various bone segments of 2 years and 8 months old boy (patient 10). **a** DWIBS (anterior). **b** ^123^I-MIBG scintigraphy/SPECT (anterior). The MIP of ^18^F-FDG PET (**c**) and DWIBS (**d**). Long arrows show the original lesion (**a**–**d**). Arrowheads show false-positive images (**a, c, d**)

The example false-positive images in various bone segments by DWIBS are shown along with the negative results by ^18^F-FDG PET/CT, ^123^I-MIBG scintigraphy/SPECT-CT, and bone scintigraphy/SPECT in Figs. 1, 2, 4, 5 and 6. Diffuse intensive false-positive signals by DWIBS are seen in pelvic bones (iliac and sacral bones) (Fig. 4a). Faint diffuse false-positive signals are seen in various bones (Figs. 5, 6).

## Discussion

There have so far been many reports on the utility of ^18^F-FDG PET or ^18^F-FDG PET/CT to pediatric neuroblastoma [4–8]. All these studies showed that ^18^F-FDG PET can provide effective information on the diagnosis and prognosis for neuroblastoma patients. Comparison was made between ^18^F-FDG PET and ^123^I-MIBG scintigraphy in detecting neuroblastoma lesions [5, 6]. It was shown that both ^18^F-FDG PET and ^123^I-MIBG scintigraphy were complementarily efficient in detecting neuroblastoma lesions [5], or that ^18^F-FDG PET was more sensitive and specific than ^123^I-MIBG scintigraphy in detecting neuroblastoma lesions [6]. It was also shown that ^18^F-FDG PET was superior to CT in detecting distant lymph node metastasis and to bone scintigraphy in detecting bone metastasis in neuroblastoma [7]. In the present study, the sensitivity of ^123^I-MIBG scintigraphy/SPECT-CT in detecting lymph node and bone metastases were compatible with those of ^18^F-FDG PET/CT.

In the present study, the specificity of ^18^F-FDG PET/CT for detecting bone metastasis (79/108, 73.1%) was lower than that for detecting lymph node metastasis (74/75, 98.7%) from neuroblastoma, because the presence of false-positive cases in bone segments without metastasis (29/108, 26.9%) was more than that in lymph node regions without metastasis (1/75, 1.3%) (Tables 4, 5, 6). However, the efficiency of ^18^F-FDG PET/CT for detecting bone metastasis from neuroblastoma were compatible with those reported previously [4–8]. In 7 of the 13 study patients in the present study, assessments by various imaging modalities were performed at least 13 days after starting the treatment including chemotherapy. It has been well known that anticancer chemotherapy stimulates uptake of FDG in bone marrow. The presence of such a number of false-positive findings as 26.9% in bone segments without metastasis may be due to this fact.

In pediatric patients, DWIBS has been confirmed, based on ADC values of the original lesions, to discriminate between benign and malignant tumors including neuroblastic tumors [15–19] and also to efficiently judge response to chemotherapy in pediatric neuroblastoma [20]. In adult patients, however, there have been reports indicating that signal intensity of the lesion-to spinal cord ratio (LSR) measured on high b-value DWIBS is more useful than ADC for differentiating between benign and malignant lung nodules [23, 24]. It was found that LSR of cancer nodules was significantly higher than that of benign lesions, while there was no significant difference between them in ADC [23].

In the present study, to evaluate diagnostic performance of whole-body DWIBS in detection of lymph node and bone metastases from pediatric neuroblastoma, signal intensities of the metastatic lesions which were visually stronger than those of skeletal muscles as the reference standard were judged as positive findings. Since there were many false-positive findings in DWIBS images of spines, skeletal muscles were adopted as the reference standard in place of spinal cord [23, 24]. The sensitivity, specificity, overall accuracy, PPV, and NPV of whole-body DWIBS for detecting bone metastasis from neuroblastoma were 94.7, 24.0, 53.0, 46.4, and 86.7%, respectively, whereas those for detecting lymph node metastasis were 94.7, 85.3, 87.2, 62.1, and 98.5%, respectively (Tables 5, 6). The low specificity, overall accuracy, and PPV of whole-body DWIBS for detecting bone metastasis was due to a high incidence of false-positive findings (82/108, 75.9%) (Table 4). At whole-body DWIBS, there were 5 false-positive findings within lumbar spine in 5 patients who had no metastases in lumbar spine (Tables 3, 8). The results indicate that lumbar spines of all the 5 patients who had no metastasis in lumbar spine had false-positive images. And also at whole-body DWIBS, there were 2 false-positive findings both within left and right pelves of 3 patients, 2 of whom had no metastasis in both left and right pelves and 1 had no metastasis in left pelvis (Tables 3, 8).

There was a report which suggests that DWIBS may be used to evaluate the localization of parenchymal neoplasms but is less efficient in characterizing lymph node and bone metastases [25]. In 2011, Müller et al. [26] reported that all children had areas of high signal both within lumbar spine and pelvic skeleton when 42 healthy children (24 boys) underwent DWIBS of the abdomen and pelvis. Children to be studied were aged 2 months to 16 years (median age, 9.6 years), all Caucasian living in the Tomsø district in northern Norway. Children younger than 5 years of age had homogeneous high signal in the lumbar vertebrae, children between 5 and 10 years of age showed a mixed pattern with signal both in the periphery and in the center of vertebrae, and in children older than 10 years of age the high signal was in the periphery of lumbar vertebrae. In the pelvis, no specific distribution patterns of high signal were seen, except for the growth plates, all of which showed high signal of slightly varying intensity. No high signal was found in any proximal femoral epiphyses. The younger children had more extensive distribution of high signal in pelvic skeleton and the high signal tended to reduce with age. The change in quantity of high signal in lumbar vertebrae and pelvis is possibly related to bone marrow conversion from red bone marrow to fatty yellow bone marrow.

In the present study, false-positive findings at whole-body DWIBS were seen in all bone segments (Table 8). Typical patterns of signal distribution in false-positive findings were high diffuse signal in pelvic skeleton and faint diffuse signal in the other bone segments (Figs. 4, 5, 6). All the 13 pediatric patients studied in the present study were younger than 5 years of age. Patterns of false-positive findings in pelvis at DWIBS were very similar to high signals in pelvis of healthy children reported by Müller et al. [26], whereas faint diffuse signals as false-positive findings in lumbar spine seen in the present study were different from typical patterns found in lumbar spine of healthy children reported by them [26]. True reason for the differences in the patterns of false-positive signals at DWIBS in lumbar spine between our findings and those reported by them is unknown. However, the results of the present study provide the same conclusion as theirs that DWIBS should be carefully used for cancer staging in children because of its high incidence of false-positive findings in skeletons.

There were some limitations in this study. One major limitation was that the quantitative analysis using ADC or LSR values was not performed for comparison of true- and false-positive foci at whole-body DWIBS. This objective awaits further investigation including readout-segmented EPI which is the technique for DWI capable of minimizing artifacts [27]. Besides, no special consideration was paid on the problem that two radiologists participated in this study determined the reference standard and evaluated the images.

## Conclusion

^18^F-FDG PET/CT exhibits good sensitivity, specificity, overall accuracy, PPV, and NPV for detecting lymph node and bone metastases from pediatric neuroblastoma. In contrast, the specificity, overall accuracy, and PPV of whole-body DWIBS are lower than those of ^18^F-FDG PET/CT because of a high incidence of false-positive findings at whole-body DWIBS particularly for detecting bone metastasis, whereas whole-body DWIBS shows a similar level of sensitivities for detecting lymph node and bone metastases to those of ^18^F-FDG PET/CT. DWIBS should be carefully used for cancer staging in children because of its high incidence of false-positive findings in skeletons.
